# No Effects of Mental Fatigue and Cerebral Stimulation on Physical Performance of Master Swimmers

**DOI:** 10.3389/fpsyg.2021.656499

**Published:** 2021-07-05

**Authors:** Eduardo Macedo Penna, Edson Filho, Bruno Teobaldo Campos, Renato Melo Ferreira, Juliana Otoni Parma, Guilherme Menezes Lage, Victor Silveira Coswig, Samuel Penna Wanner, Luciano Sales Prado

**Affiliations:** ^1^GET/UFPA – Grupo de Estudos em Treinamento Físico e Esportivo, Universidade Federal do Pará, Castanhal, Brazil; ^2^LAFISE – Laboratório de Fisiologia do Exercício, Universidade Federal de Minas Gerais, Belo Horizonte, Brazil; ^3^Wheelock College of Education and Human Development, Boston University, Boston, MA, United States; ^4^LAQUA – Laboratório de Atividades Aquáticas, Universidade Federal de Ouro Preto, Ouro Preto, Brazil; ^5^NNeuroM – Núcleo de Neurociências do Movimento Humano, Universidade Federal de Minas Gerais, Belo Horizonte, Brazil; ^6^CTE- Centro de Treinamento Esportivo/UFMG, Belo Horizonte, Brazil

**Keywords:** mental fatigue, swimming, transcranial direct current stimulation, master athletes, endurance

## Abstract

**Background:** Mental fatigue is a psychobiological state caused by extended periods of cognitive effort, and evidence suggests that mentally fatigued athletes present impaired physical performance. Different ergogenic aids have been proposed to counteract the deleterious effects of mental fatigue, but whether brain stimulation can counteract mental fatigue is still unknown. This scenario is even more obscure considering the effects of these interventions (mental fatigue induction and brain stimulation) in a very experienced population consisting of master athletes.

**Method:** Ten master swimmers (30 ± 6 years old and 14 ± 8 years of experience) participated in the study. They underwent four experimental conditions before an 800-m freestyle test: mental fatigue with brain stimulation; mental fatigue without brain stimulation; absence of mental fatigue with brain stimulation; and absence of mental fatigue and no brain stimulation. Mental fatigue was induced by a cognitively demanding Stroop Color Test, whereas stimulation was applied on the temporal cortex. After that, the athletes swan 800 m as fast as possible and provided their ratings of perceived exertion (RPE) every 200 m.

**Results:** Mental fatigue was effectively induced, as evidenced by a greater fatigue perception and more errors in the last blocks of the cognitive task. Mental fatigue induction did not influence performance (time to complete the swimming trial) and RPE. Similarly, brain stimulation failed to change these two parameters, regardless of mental fatigue induction.

**Conclusion:** The prolonged physical performance of experienced master athletes is not influenced, under the present conditions, by mental fatigue induction, cerebral stimulation, and their association.

## Introduction

Mental fatigue refers to a psychobiological state caused by prolonged and/or intense periods of cognitive exertion and is characterized by changes in behavioral, cognitive, and physiological responses (Cutsem et al., 2017). Mental fatigue has been shown to reduce aerobic performance, particularly in individual sports (Mac Mahon et al., 2014; Penna et al., 2018a,b; Pires et al., 2018; Staiano et al., 2018), although some studies have failed to demonstrate reduced performance in mentally fatigued athletes (Martin et al., 2016; Filipas et al., 2018; Salam et al., 2018; Silva-Cavalcante et al., 2018; Holgado et al., 2020).

The mechanisms linking mental fatigue to performance are not yet fully understood. Physiological measures associated with aerobic performance, such as the heart rate, blood lactate accumulation, and oxygen consumption, tend not to change due to mental fatigue (Marcora et al., 2009). However, perceptual changes, such as ratings of perceived exertion (RPE) and perceived exercise rewards (Schiphof-Godart et al., 2018), are frequently observed in mentally fatigued athletes. Therefore, it has been proposed that long periods of highly demanding cognitive activity may increase extracellular brain adenosine concentrations, which in turn may negatively affect RPE and reduce an individual’s ability to make substantial efforts during an exercise (Martin et al., 2018).

In this sense, different acute ergogenic strategies have been proposed to counteract the adverse effects of mental fatigue in different sports and populations, including acute caffeine consumption (Azevedo et al., 2016; Meeusen et al., 2017; Franco-Alvarenga et al., 2019). Notably, these studies have shown that caffeine attenuated the abnormal increase in RPE under mentally fatiguing conditions. In addition to caffeine, transcranial direct current stimulation (tDCS), which consists of applying a weak electrical current on the scalp to alter neuronal excitability for extended periods (Colzato et al., 2016), has been shown to improve physical performance (Pollastri et al., 2021) and reduce RPE in predominantly aerobic activities (Okano et al., 2015; Lattari et al., 2016; Valenzuela et al., 2018). The potential mechanism underlying the ergogenic effect of tDCS could be increased cortical excitability in specific brain areas targeted by anodal stimulation (Kaushalya et al., 2020). However, some evidence provides no strong support for beneficial effects on performance (Holgado et al., 2019). These contrasting results could rely on methodological differences, such as neuromodulation focality (Pollastri et al., 2021) and the task analyzed (e.g., time to exhaustion or endurance time trial; Kaushalya et al., 2020).

An important characteristic that must be considered concerning tDCS is the target area. Studies have stimulated the left temporal cortex, which likely increased neuronal excitability of the insular cortex and influenced collateral regions, such as the right temporal cortex and frontal lobe (Okano et al., 2015; Barwood et al., 2016). The rationale for stimulating the insular cortex relies on this region’s involvement in the awareness of body subjective feelings related to the athletes’ perceptions of physical exertion during exercise (Williamson et al., 2017).

In the sport context, master athletes represent a particular type of athlete whose participation in competitions has increased substantially in the past decades (Lepers and Stapley, 2016). Each sport has its own rule for classifying master athletes; in swimming, competition for these athletes begins when they become 25 years old (Reaburn and Dascombe, 2008). Notwithstanding the decrease in physical performance due to aging being well documented (Reaburn and Dascombe, 2008) and some psychological variables (such as motivation) already been investigated in this population (Hodge et al., 2008), psychophysiological determinants of performance (such as RPE and pacing) as well as the response of this population to different ergogenic resources related to physical performance are less studied.

In summary, mental fatigue often impairs physical performance, mainly through abnormal perceptual responses during exercise. Conversely, brain stimulation may improve performance by reducing RPE in some contexts. However, whether brain stimulation can counteract the (possible) impaired performance in mentally fatigued athletes is still unknown. This scenario is even more obscure considering the effects of these interventions (mental fatigue induction and brain stimulation) in master athletes. In the present study, we aimed to compare the isolated and additive effects of mental fatigue and brain stimulation on the physical performance of master swimmers. It was hypothesized that mental fatigue and brain stimulation would, respectively, reduce and improve performance and that brain stimulation would attenuate the impairment caused by mental fatigue.

## Materials and Methods

The experimental task consisted of a swimming test, during which the participants swam a distance of 800 m in an Olympic-size pool (50 m in length) as fast as possible. A within-subject design with four randomized experimental conditions was used. Prior to the swimming tests, the participants were randomly assigned (using the Web site tool www.randomizer.org) to one of the 24 possible sequences of the following four experimental manipulations: mental fatigue with brain stimulation (MF + BS); mental fatigue without brain stimulation (MF + SHAM); absence of mental fatigue with brain stimulation (CONT + BS); and absence of mental fatigue and no brain stimulation (CONT + SHAM).

**Figure 1:**
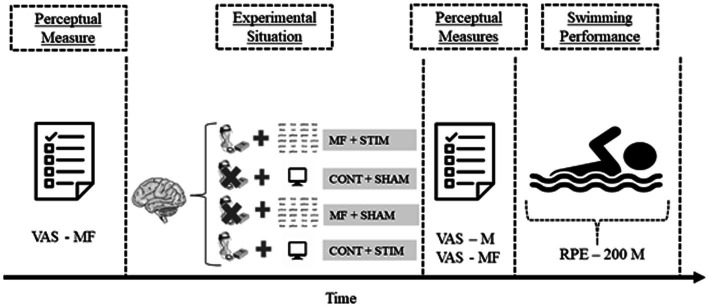
Experimental design. CONT + BS, control procedure plus brain stimulation; CONT + SHAM, control procedure plus SHAM stimulation; MF + BS, mental fatigue plus brain stimulation; MF + SHAM, mental fatigue plus SHAM stimulation; RPE, rating of perceived exertion; VAS – M, visual analog scale for motivation; and VAS – MF, visual analog scale for mental fatigue.

Before each experimental condition, the participants were briefed on the data collection procedures. Of note, to prevent demand effects and confirmation bias, information about the order of experimental conditions was concealed from the participants. First, the participants completed a visual analog scale (VAS) to rate their baseline mental fatigue perception, and then, the brain stimulation device was assembled, followed by the protocol for inducing mental fatigue or control manipulation. Next, the volunteers completed the perceptual scales (i.e., mental fatigue perception and motivation for subsequent physical testing), which corresponded to perception at post-mental fatigue or post-control manipulation. After completing the scales, the volunteers were taken to the pool (lane 1 or 8) to perform the swimming test. They were accompanied by the leading researcher while swimming and were asked to verbalize their RPE score every 200 m. A summary of the experimental design is presented in Figure 1.

### Participants

Ten male master swimmers (age 30 ± 6 years) with 14 ± 8 years of experience with regular training and competitions took part in this study. All swimmers trained 3–4 days per week for approximately seven weekly hours of training. At the time of the study, all participants were competing at the highest national level in their respective age groups.

The volunteers signed an informed consent form, which was approved by the local Ethics Committee of the leading author’s university (protocol number 69475417.7.0000.5149). Data concerning the mental fatigue-induced decrement in physical performance from Staiano et al. (2018) were used to calculate the sample size based on the following specifications: *α* = 0.05, (1–*β*) = 0.80, and effect size = 0.93.

### Instruments

#### Mental Fatigue Induction Protocol

Similar to previous research in sports, the paper version of the Stroop Color Test was employed to induce mental fatigue (Smith et al., 2016; Penna et al., 2018b; Campos et al., 2019). The 45-min test consisted of presenting words colored in green, red, blue, and yellow. The participants were asked to verbalize the color of the word (i.e., ink color) rather than the written word presented to them. The number of correct words and mistakes were recorded by the leading author. For each mistake made, the participants were asked to come back to the beginning of the line where the mistake occurred. The control trial involved watching a 45-min video identified as emotionally neutral in a pilot test (induced no changes in the heart rate, heart rate variability, or mood).

#### Visual Analog Scale

As suggested (Smith et al., 2019), perceived motivation and mental fatigue were measured using a 100-mm VAS anchored by the expressions “not at all” and “maximum.” The distance between the starting point and the marking made by the participants was measured with a ruler, and the values obtained were analyzed as arbitrary units (a.u.). These manipulation checks were carried out to verify whether the athletes (1) performed the physical test under similar conditions of motivation and (2) perceived the cognitive test as mentally fatiguing, and in fact, experienced some mental fatigue level.

#### Cerebral Stimulation Protocol

The brain stimulation method used was the tDCS (stimulator model 1300A, Soterix Medical, New York City, United States), which is a noninvasive, reversible, and safe technique (Gazerani, 2017). Each electrode had a surface area of 35 cm^2^. The intensity used was 2.0 mA as previously used in exercise science studies (Angius et al., 2015, 2016).

The stimulation was applied with two electrodes covered by sponges soaked in a saline solution to avoid heat transfer to the scalp. An anode (positive) electrode was placed over the left temporal cortex (T3 area of the international 10–20 EEG system), which is the excited region, according to the method used by Okano et al. (2015). The cathode (negative) electrode was placed over the ipsilateral shoulder, as suggested by Angius et al. (2016). When stimulation was applied, subjects were stimulated over the temporal cortex during the last 30 min of the Stroop Color Test or control situation (film; SHAM). Similarly, during both SHAM conditions, the participants underwent the same procedures as in conditions with stimulation (the same equipment, application site, and current intensity were used, but the stimulation was maintained for only 30 s and then gradually withdrawn without the knowledge of the volunteers). This strategy was designed to keep volunteers blinded from the experimental procedure since most individuals report an itching sensation only at the beginning of the current application. The equipment was kept in position until the Stroop Color Test (or control procedure) was completed.

#### Physical Performance Measure

As physical performance measures, the times taken to complete each 50 m (pacing) and the entire swimming trial (i.e., 800 m) were recorded.

#### Rating of Perceived Exertion

Similar to a previous study (Penna et al., 2018b), participants were asked to verbalize their perceived exertion relative to current performance every 200 m, using a previously validated 10-point scale (Foster et al., 2001). According to the volunteers’ breathing laterality, score verbalization was made without interruptions in swimming at each 200-m arrival or departure. RPE was measured during the test to verify whether the athletes perceived their physical effort differently between experimental situations. As previously reported (Marcora et al., 2009), mental fatigue could induce higher RPE during prolonged exercise. Indeed, taking Marcora’s psychobiological model to explain exercise performance into account, RPE and motivation are considered to influence voluntary control, ultimately determining the neural activation of locomotor muscles (Marcora, 2008).

### Statistical Analysis

Two-way repeated-measures ANOVAs (main factors: mental fatigue and brain stimulation) were used to compare the physical performance (800-m swimming time), RPE, pacing, motivation, and the changes (post- minus pre-treatment) in mental fatigue. The number of words answered and errors in the Stroop Color Test were also analyzed using two-way repeated-measures ANOVAs (main factors: condition and test duration). A one-way repeated-measures ANOVA was conducted to analyze whether the order of the experimental trials affected physical performance. All statistical analyses were performed using the GraphPad Prism 7.0 software, and the significance level adopted was *α* < 0.05.

## Results

### Manipulation Check – Mental Fatigue

**Figure 2:**
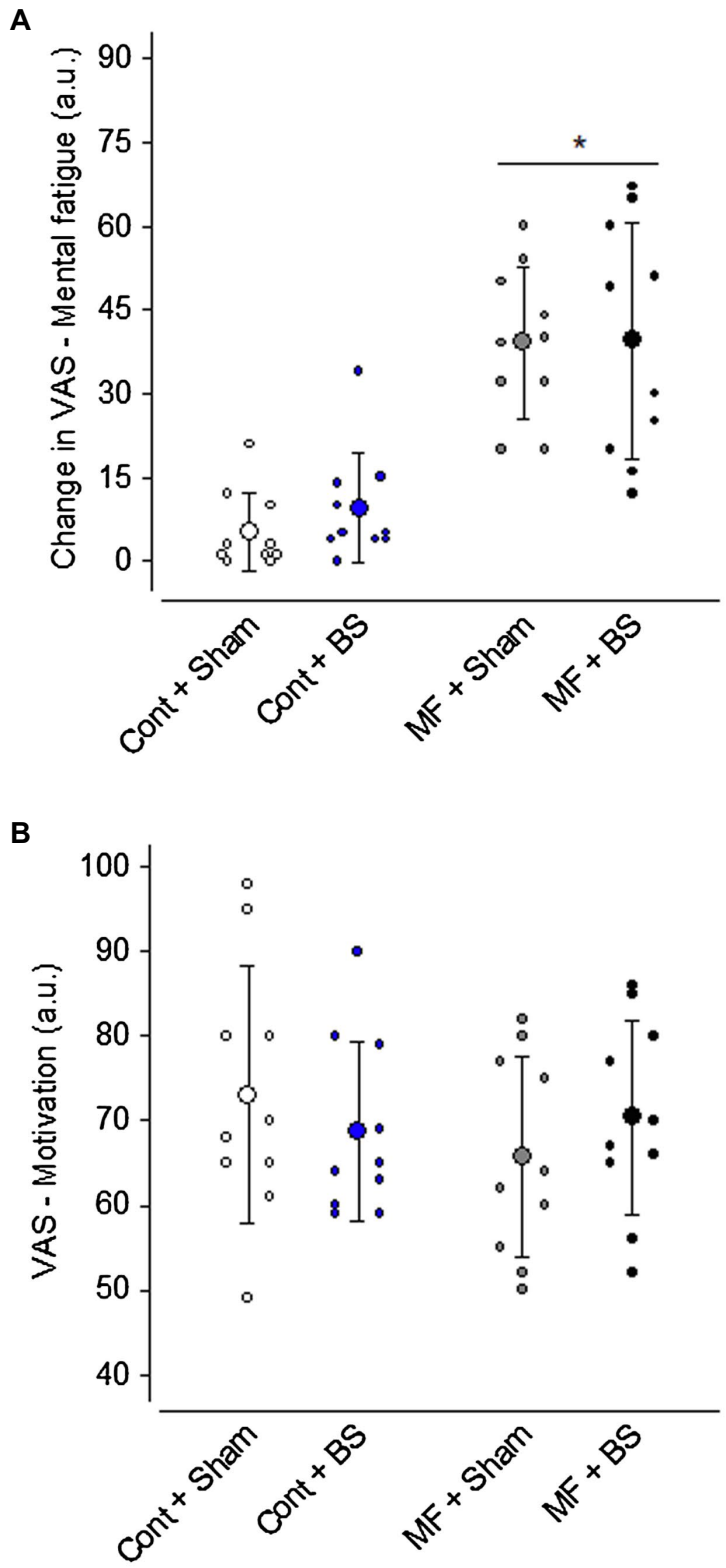
Change in the perception of mental fatigue from pre-treatment to post-treatment **(A)** and motivation for the subsequent swimming task **(B)**. Data are expressed as mean ± standard deviation, and the scattered dots represent the individual data. * means a significant main effect of mental fatigue (*p* < 0.05).

As expected, the perception of mental fatigue (i.e., the change between post- and pre-treatment) was higher in situations where the Stroop test was administered (MF + BS: 39 ± 21 a.u., MF + SHAM: 39 ± 13 a.u.) than in control situations [CONT + BS: 9 ± 8 a.u., CONT + SHAM: 5 ± 6 a.u.; *F*(1,36) = 52.65, *p* < 0.001, *ηp*^2^ = 0.59; Figure 2A]. There was no main effect of brain stimulation on the perception of mental fatigue [*F*(1,36) = 0.284, *p* = 0.59, *ηp*^2^ = 0.007], nor an interaction between the two factors. Therefore, the cerebral stimulation did not affect how participants rated their perceived mental fatigue after the Stroop test.

Regarding motivation (Figure 2B), no differences were identified between the mental fatigue and control conditions [MF + BS: 70 ± 11 a.u., MF + SHAM: 65 ± 10 a.u., CONT + BS: 68 ± 10 a.u., CONT + SHAM: 73 ± 15 a.u.; *F*(1,36) = 0.541, *p* = 0.46, *ηp*^2^ = 0.01] or between with and without brain stimulation [*F*(1,36) = 0.002, *p* = 0.95, *ηp*^2^ < 0.001]; also, there was no interaction between the two factors [*F*(1,36) = 1.304, *p* = 0.26, *ηp*^2^ = 0.01]. Therefore, the volunteers initiated all experimental conditions with similar motivation.

### Cognitive Performance During Stroop Color Test

**Figure 3:**
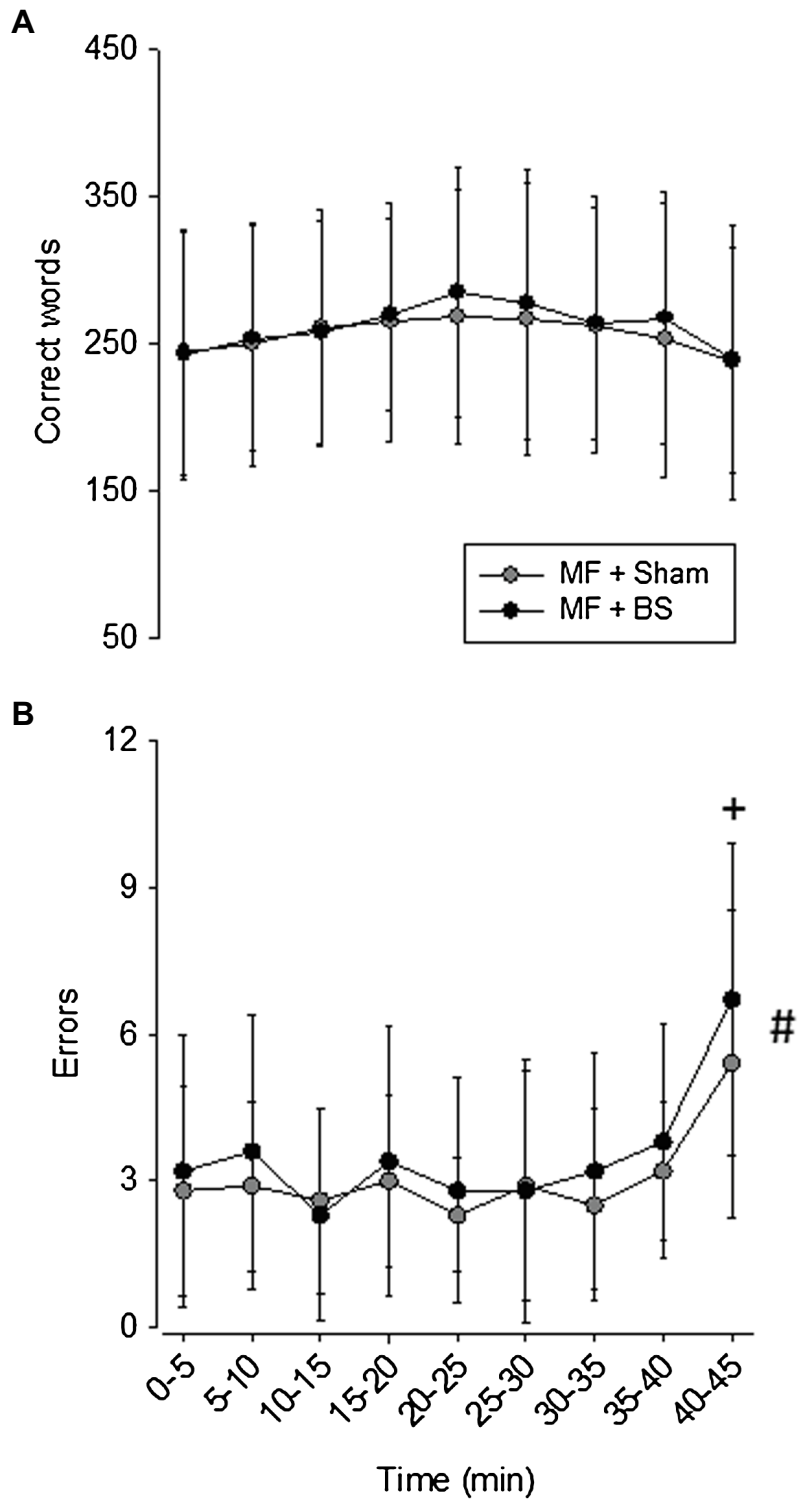
Number of correct words **(A)** and errors **(B)** during the 45-min Stroop Color Test. Data are expressed as mean ± standard deviation. + means a significant difference from all other time points for both conditions (*p* < 0.05). # means a significant main effect of time (*p* < 0.05).

To better understand the induction of mental fatigue and the effects of transcranial stimulation on this induction, we recorded the number of correct words and the number of errors the participants have made during the Stroop Color Test (Figure 3). Regarding the number of correct words, no significant main effects of test duration [*F*(8,81) = 0.639, *p* = 0.74, *ηp*^2^ = 0.003] and brain stimulation [*F*(8,81) = 0.257, *p* = 0.61, *ηp*^2^ = 0.06] and no significant interaction between these factors were observed [*F*(8,81) = 2.106, *p* = 0.99, *ηp*^2^ = 0.004; Figure 3A]. Concerning the number of errors (Figure 3B), no main effect of brain stimulation [*F*(8,81) = 1,888, *p* = 0.17, *ηp*^2^ = 0.02] and no significant interaction between time and brain stimulation were observed [*F*(8,81) = 0.209, *p* = 0.98, *ηp*^2^ = 0.02]. However, a significant main time effect was identified [*F*(8,81) = 4.057, *p* = 0.00198, *ηp*^2^ = 0.30], indicating that participants committed more errors during the last 5 min compared to all other 5-min blocks.

### Physical Performance

**Figure 4:**
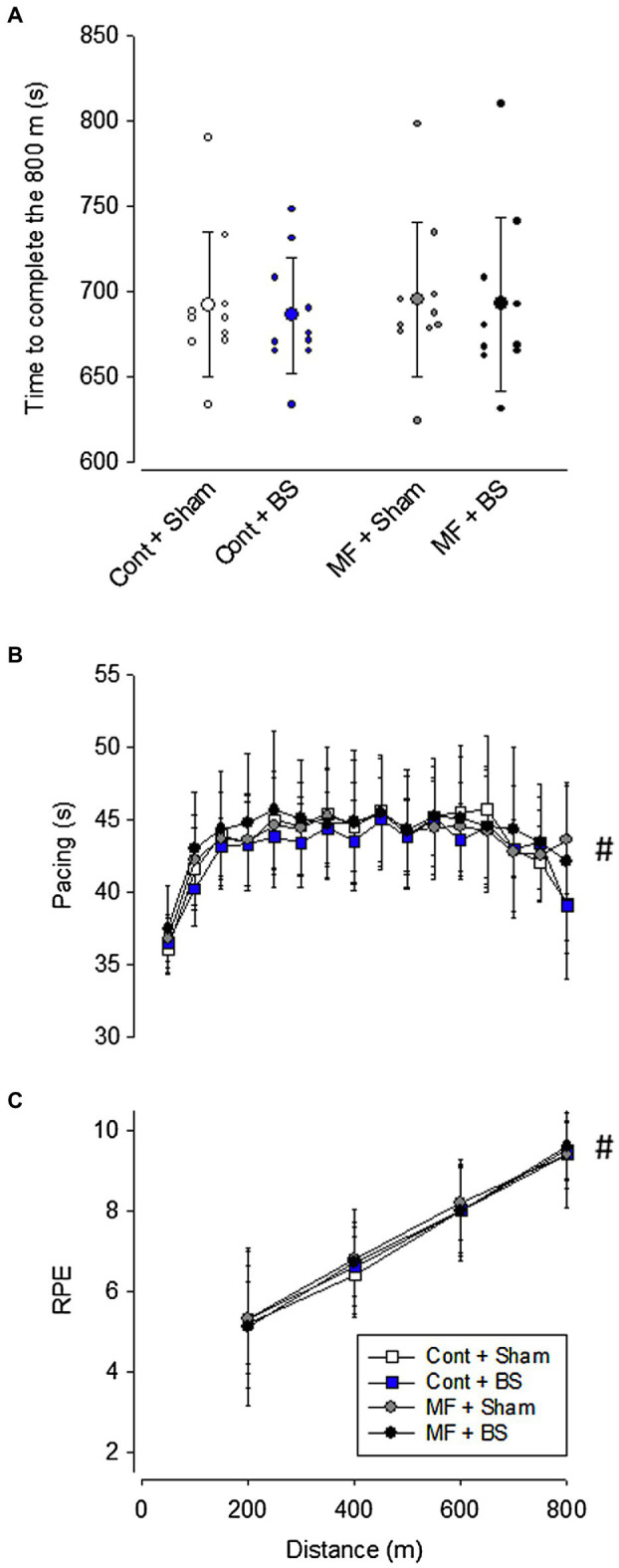
Time to complete the 800 m of swimming **(A)**. Data are expressed as mean ± standard deviation, and the scattered dots represent the individual data. Pacing strategy **(B)** and subjective rating of perceived exertion **(C)** of the participants throughout the swimming trial. Data are expressed as mean ± standard deviation. # means a significant main effect of distance (*p* < 0.05).

The order of the experimental trials did not affect physical performance [*F*(3,36) = 0.03568, *p* = 0.99], indicating no influence of learning or familiarization in our results. Physical performance was similar across all experimental situations (MF + BS: 692 ± 50 s, MF + SHAM: 695 ± 45 s, CONT + BS: 686 ± 34 s, CONT + SHAM: 692 ± 42 s; Figure 4A). There was no significant main effect of mental fatigue [*F*(8,81) = 0.500, *p* = 0.49, *ηp*^2^ = 0.003] and brain stimulation [*F*(8,81) = 0.260, *p* = 0.62, *ηp*^2^ = 0.002], as well as no significant interaction between these two treatments [*F*(8,81) = 0.076, *p* = 0.78, *ηp*^2^ < 0.001]. Similarly, no differences were identified in pacing (Figure 4B) between the experimental conditions [*F*(3,36) = 0.271, *p* = 0.84, *ηp*^2^ = 0.01], nor an interaction between mental fatigue and brain stimulation [*F*(45,540) = 1.182, *p* = 0.20, *ηp*^2^ = 0.03]. However, there was a significant main effect of distance [*F*(15,540) = 41.980, *p* = 0.01, *ηp*^2^ = 0.27], indicating that athletes regulated their pacing differently over the 800-m swimming, with the highest speeds being observed at the first 50 m (likely due to the diving start). Regarding RPE scores (Figure 4C), no interaction between mental fatigue and brain stimulation [*F*(9,108) = 0.219, *p* = 0.99, *ηp*^2^ = 0.01], and no differences between the experimental situations were observed [*F*(3,36) = 0.041, *p* = 0.98, *ηp*^2^ < 0.001]; as expected, the main effect of distance reached statistical significance [*F*(3,108) = 210.800, *p* = 0.01, *ηp*^2^ = 0.85]. Altogether, these data suggest that mental fatigue was not sufficiently strong to impair physical performance and that brain stimulation was not efficient in improving physical performance when participants were not mentally fatigued.

## Discussion

The present study aimed to compare the isolated and additive effects of mental fatigue and brain stimulation on the physical performance and perceptual responses of master athletes during an 800-m swimming test. Contrary to our hypotheses, neither mental fatigue nor brain stimulation influenced the athletes’ physical performance and RPE in the present conditions.

### Manipulation Check – Mental Fatigue

The protocol used for inducing mental fatigue in the present study was effective. Specifically, the results (i.e., an increase in perceived mental fatigue and more errors in the Stroop Color Test) indicate that the athletes experienced some level of mental fatigue after the protocol. As described by Cutsem et al. (2017), mental fatigue can manifest itself as subjective (measured through self-reports), behavioral (measured through reaction time or performance on a cognitive task), or physiological responses (measured by reducing brain activation, for example). Importantly, no possible changes in subsequent physical performance could have been credited to changes in motivational levels as, across conditions, athletes started swimming with similar motivation.

Furthermore, our analysis revealed that brain stimulation did not reduce the perception of mental fatigue, nor did it improve cognitive performance during the Stroop Color Test. In the present study, we stimulated the temporal cortex, which is associated with long-term memory management (Simons and Spiers, 2003) and the awareness of subjective feelings of the body; these feelings are related to athletes’ perceived physical exertion during dynamic exercises (Williamson et al., 2017). Although long-term memory is not widely required by the Stroop Color Test, which is closely related to the anterior cingulate cortex activity (Laird et al., 2005), there was an expectation that tDCS would have a relatively broad effect (Filmer et al., 2014). However, this expectation was not confirmed, likely because the diffuse effect of the tDCS was not sufficient to induce behavioral changes (changes in performance) in the present conditions. In this sense, future studies using the novel high-definition (HD)-tDCS technique (Villamar et al., 2013) may help avoid the undesired diffuse effect induced by tDCS.

### Performance and Mental Fatigue

Mental fatigue did not negatively influence performance, nor did pacing or RPE (Figures 4A–C). While performing a prior cognitive task has been shown to reduce physical performance (McMorris et al., 2018), the experimental evidence related to elite athletes remains inconclusive (Russell et al., 2019). To this extent, previous research has not shown performance degradation in a time-to-exhaustion test in cycling (Martin et al., 2016; Salam et al., 2018; Clark et al., 2019) and football (Smith et al., 2016). It has been suggested that elite athletes are more resilient (e.g., more resilient in keeping their cognitive skills) than amateur or non-athlete individuals (Faubert, 2013), which explains the lesser (or nonexistent) effect of mental fatigue on performance for this elite population. It is also important to highlight that athletes’ experience might have a moderating effect on mental fatigue. Specifically, Penna et al. (2018b) examined young swimmers and found that mental fatigue, as induced by a Stroop Color Test, had a negative effect on performance; however, the present findings obtained with experienced master athletes contrast with the previous findings concerning young swimmers.

The present results corroborate previous research on the effects of mental fatigue on pacing during self-regulated activities (Pageaux et al., 2014; Pires et al., 2018). Even in studies where a change in performance in mentally fatiguing situations has been reported, changes in pacing are not commonly observed (Pires et al., 2018). In other words, even when performance changes (slower running or swimming speeds), the pacing seems to be unaffected by mental fatigue (Pageaux and Lepers, 2018), thus suggesting that pacing may be a more persistent cognitive domain.

Contrary to our hypothesis, RPE was not altered by the experimental induction of mental fatigue before the 800-m swimming. This result agrees with the observation that athletes’ physical performance did not differ between the experimental conditions. It is noteworthy mentioning that our athletes were not exclusively dedicated to the sport, meaning that they should constantly balance the physical and psychophysiological stress of training routine with other work and family-related stressors, making them more resistant to mental fatigue and the resulting RPE impairment (Martin et al., 2016).

### Cerebral Stimulation

The fact that the performance was not impaired after the mental fatigue protocol (MF + SHAM conditions) limits the discussion about tDCS being able to counteract the deleterious effect of mental fatigue on performance. However, contrary to our hypothesis and the results of previous studies (Okano et al., 2015; Vitor-Costa et al., 2015; Pollastri et al., 2021), tDCS did not increase physical performance (as evidenced by comparing CONT + BS and CONT + SHAM conditions) in master swimmers even when they were not mentally fatigued.

Regarding the stimulated brain area, temporal cortex activity is associated with cardiac autonomic control (Hilz et al., 2002; Okano et al., 2015), as well as emotional regulation and perception of effort (Williamson et al., 2017). Therefore, since there was no measurement of cardiac autonomic control in the present study, it can be speculated that if stimulation of the temporal cortex modulated cardiac autonomic control through late vagal withdrawal, as demonstrated by Okano et al. (2015), this modulation might not have been sufficiently strong to reflect a better performance in master athletes. In addition, our sample consisted of athletes with extensive experience in regular physical activity (over 14 years of regular training), and they may possess an optimized function of the temporal cortex region due to regular physical activity (Bugg and Head, 2011), thus nullifying the possible beneficial effects of tDCS due to a ceiling effect.

The application of tDCS during the prolonged Stroop Color Test also failed to improve cognitive performance given that both the number of correct words and errors were not different between the situations (i.e., MF + BS versus MF + SHAM). This result reinforces the importance of the area stimulated, as studies involving similar stimuli in other brain areas (e.g., the supplementary motor area and the lateral dorsal prefrontal cortex; see Hsu et al., 2011; Loftus et al., 2015) have led to performance improvements in inhibitory control, which is the main executive component recruited by the Stroop Color Test. Therefore, temporal cortex stimulation does not appear to modulate significantly the performance in tasks requiring inhibitory control.

This study has some limitations that need to be reported. Although we have followed the stimulation site proposed by Okano et al. (2015), it was not possible to apply computational techniques or influence maps to ensure that the temporal cortex was indeed stimulated. Moreover, the lack of ecological validity of the task used for inducing mental fatigue (i.e., the Stroop Color Test) hinders the application of the theoretical model in real-world training and competitions.

### Conclusion

In conclusion, a mentally demanding task before an endurance (800 m) swimming test did not impair the performance of master athletes. Additionally, cerebral stimulation of the temporal cortex did not improve the performance of these athletes even when mental fatigue was not present. These results indicate that master athletes may regulate their physical performance differently from other athletes, thus challenging the notion that brain stimulation and mental fatigue determine endurance, at least in the population investigated in the present study.

## Data Availability Statement

The raw data supporting the conclusions of this article will be made available by the authors, without undue reservation.

## Ethics Statement

The studies involving human participants were reviewed and approved by Universidade Federal de Minas Gerais Ethics Committee. The patients/participants provided their written informed consent to participate in this study.

## Author Contributions

EP, EF, GL, VC, SW, and LP designed the experiment. EP, BC, RF, and JP carried out the testing of participants and drafted the manuscript. All authors contributed to the critical revision of the manuscript and approved the final version.

### Conflict of Interest

The authors declare that the research was conducted in the absence of any commercial or financial relationships that could be construed as a potential conflict of interest.
